# Optimizing Josephson Junction Reproducibility in 30 kV E-Beam Lithography: An Analysis of Backscattered Electron Distribution

**DOI:** 10.3390/nano14090783

**Published:** 2024-04-30

**Authors:** Arthur M. Rebello, Lucas M. Ruela, Gustavo Moreto, Naiara Y. Klein, Eldues Martins, Ivan S. Oliveira, João P. Sinnecker, Francisco Rouxinol

**Affiliations:** 1 Coordenação de Matéria Condensada, Física Aplicada e Nanociência (COMAN), Centro Brasileiro de Pesquisas Físicas (CBPF), Rio de Janeiro 22290-180, RJ, Brazil; artrebello@cbpf.br (A.M.R.); naiaraklein@gmail.com (N.Y.K.); ivan@cbpf.br (I.S.O.); 2 Quantum Device Physics Laboratory, Universidade Estadual de Campinas (Unicamp), Instituto de Física Gleb Wataghin (IFGW), Campinas 13083-859, SP, Brazil; lucasmr@ifi.unicamp.br (L.M.R.); g169380@dac.unicamp.br (G.M.); 3 Leopoldo Américo Miguez de Mello Research, Development and Innovation Center (CENPES), Rio de Janeiro 21941-915, RJ, Brazil; eldues@petrobras.com.br

**Keywords:** 30 kV e-beam, lithography, cQED, Josephson junction, nanofabrication, SQUID, quantum computing

## Abstract

This paper explores methods to enhance the reproducibility of Josephson junctions, which are crucial elements in superconducting quantum technologies, when employing the Dolan technique in 30 kV e-beam processes. The study explores the influence of dose distribution along the bridge area on reproducibility, addressing challenges related to fabrication sensitivity. Experimental methods include e-beam lithography, with electron trajectory simulations shedding light on the behavior of backscattered electrons. Wedescribe the fabrication of various Josephson junction geometries and analyze the correlation between the success rates of different lithography patterns and the simulated distribution of backscattered electrons. Our findings demonstrate a success rate of up to 96.3% for the double-resist 1-step low-energy e-beam lithography process. As a means of implementation strategy, we provide a geometric example that takes advantage of simulated stability regions to administer a controlled, uniform dose across the junction area, introducing novel features to overcome the difficulties associated with fabricating bridge-like structures.

## 1. Introduction

Superconducting quantum computing represents a frontier in contemporary physics and engineering, promising revolutionary advancements in computation [1,2,3,4], communication [5,6], and sensing [7,8,9]. Central to these technologies are Josephson junctions, which are critical components that enable the unique nonlinear properties of superconducting quantum circuits. Much progress has been made to improve the functionality and quality of Josephson junctions and their applications [10,11,12,13,14,15,16]. The fabrication process, however, remains exceedingly sensitive; even minor variations in junction area or stochastic fluctuations in oxide formation can lead to inconsistencies in the overall oxide barrier, making the reproducibility of Josephson junctions a hindering factor in the performance and scalability of quantum devices [17]. Observations from fabricating hundreds of Josephson junctions led us to examine whether variations in the geometry of these junctions could significantly influence their reproducibility. Our research indicates that backscattered electron distribution is essential to the proper formation of bridge-like structures, thereby affecting the functionality and scalability of superconducting quantum devices. Defining such correlations benefits the reliability of Josephson junctions, particularly when utilizing low-energy 30 kV electron beam lithography (EBL).

Various groups have developed distinct strategies to enhance the reproducibility of Josephson junctions using low-energy 30 kV electron beam lithography. These improvements primarily focus on variations in junction geometry and the employment of different resist stacks [18,19], which have facilitated the fabrication of superconducting qubits with relaxation and coherence times ranging from hundreds of nanoseconds to tens of microseconds [20,21,22] for diverse applications. Despite these advancements, the underlying mechanisms contributing to the enhanced reproducibility of the junctions remain ambiguous. Claims have been made regarding the influence of surface tension on the resistance [23,24], the impact of resist thickness, and charge dissipation [10,11]. To ensure the continued utility of low-energy electron beam lithography, comprehending the mechanisms affecting junction reproducibility and the role of geometry is crucial.

While the technical aspects of the 30 kV e-beam process are well documented [19], the integrity of the bridge structure is a known issue in fabricating Josephson junctions with the Dolan technique [25,26,27,28]. Some groups claim that robustness is compromised due to stress that occurs to the PMMA layer depending on the geometry used [23,24]. Others deal with this by pre-exposing the bottom resistance [18]. Our study addresses this issue by focusing on enhancing fabrication reproducibility through the strategic selection of optimal geometries that correctly engineer the doses of backscattered electrons on the bridge area. Moreover, additional teams have recognized the issue we raise in this paper; they have attempted to apply complex, commercially available 3D proximity effect correction (PEC) [29]. However, even at 100 kV, where the backscattered electron distribution is nearly homogeneous, they had to resort to manually modifying the dose in different parts of their exposure layout to achieve the desired results. We do acknowledge that even the traditional PEC [30] improved our results, and PEC was in fact used for the fabrication of all junctions in our study; nevertheless, it was not enough to yield satisfying results. In this paper, we present modifications to the fabrication methodology of Josephson junctions using 30 kV e-beam lithography, specifically employing the Dolan technique.

This manuscript is structured as follows. In Section 2, we delve into the characteristics of the samples with respect to the applied nanofabrication processes. Additionally, we elaborate on the Monte Carlo simulation process employed for simulating electron trajectories. Section 3 presents the initial simulation results, which are then correlated with experimental data. Subsequently, we analyze the simulation results further to draw additional conclusions. In Section 4, we discuss our findings, showcase their experimental applicability, and suggest potential improvements. Finally, Section 5 summarizes the main points of the manuscript.

## 2. Materials and Methods

**Figure 1:**
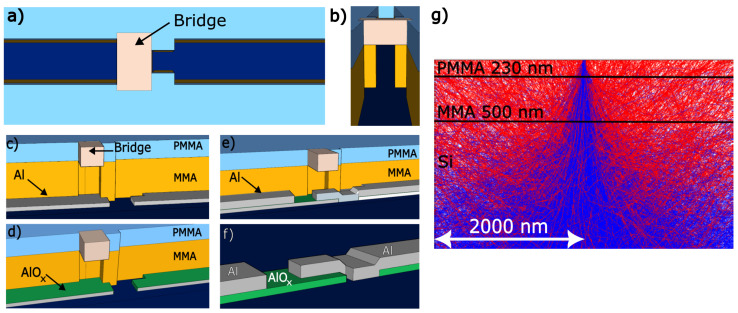
Schematic representation of the pattern transferred onto a double-layer resist stack followed by an aluminum deposition process, delineating the steps involved in fabricating Josephson junctions, alongside electron trajectory simulation within the resist layers. (**a**) Dolan Josephson junction scheme showcasing the exposed area (in dark blue) and the central bridge region (in grey). (**b**) Bird’s-eye perspective of the anticipated Josephson junction bridge structure. (**c**) Initial $30^{\circ }$ angle deposition, (**d**) oxidation phase, (**e**) subsequent $-30^{\circ }$ angle deposition. (**f**) Representation of the Josephson junction post-lift-off process, with the green coating symbolizing the $AlO_{x}$ layer. (**g**) Visualization of electron dispersion trajectories in a 230 nm PMMA layer (**top**) and a 500 nm MMA co-polymer layer (**bottom**), both situated on a silicon substrate, under the influence of a 30 kV electron beam. The trajectories of the primary electrons from the incident beam are depicted in blue, whereas the backscattered electrons are illustrated in red.

Fundamentally, the objective is to pattern the resist to form a bridge, as illustrated in Figure 1a–d. As depicted, the challenge involves employing a straight electron beam for intricate sideways sculpting beneath the resist’s top layer. This process is feasible because the electron beam generates backscattered electrons along its path, and it penetrates several micrometers into the substrate, implying backscattered can travel a long distance from the point of incidence. Backscattered electrons are highlighted in Figure 1g with red trajectories. A critical aspect of enhancing the fabrication process quality is understanding the impact of these backscattered electrons and acknowledging the variability in the sensitivity of the resist layers employed, specifically PMMA (230 nm) over MMA (500 nm).

The challenge in electron beam lithography (EBL) processes traditionally lies in optimizing them to achieve the best matching feature dimensions. For any given exposure area, a portion of the incident dose is inevitably distributed to surrounding regions, resulting in variations in the deposited dose near the beam. This phenomenon, known as the proximity effect, is mitigated using PEC techniques, which modify the dose distribution across the features to achieve a constant deposition dose within the exposed area [30]. However, these techniques primarily aim to establish a uniform dose for a given design. Here, we endeavor to advance further by examining how undercuts can be engineered to preserve the design structures of the upper resist layer. The challenge emerges from the discrepancy between the dose required to pattern the design and the dose from backscattered electrons needed to sensitize the lower resist layer, all while maintaining the integrity of the top resist layer. Given that patterning the top resist layer usually does not deposit enough dose to open the undercut in the bottom layer, an increased base dose is necessary. This adjustment, however, leads to overdosing in both the top and bottom resist layers, potentially causing deformation, weakening, and deviation from designed dimensions. While PEC helps prevent damage to the bridge structure, it is often insufficient for 30 kV applications to fully address these issues. Thus, as demonstrated in our study, a careful selection of geometric features is also crucial. We show that this approach is of utmost importance in creating reproducible structures for the fabrication of Josephson junctions.

The test structures were meticulously designed as 10 rows of 27 pads, intended to be interconnected by a Josephson junction. Each pad consists of rectangular sections measuring 200 × 200 μm^2^, with a separation of 100μm between them. These structures were fabricated by initially depositing a 100 nm thick layer of Nb or Al onto high-resistivity silicon wafers (ρ > 10 kΩ, cm). The patterning of the design was achieved using optical lithography on a Heidelberg DWL 66+, followed by the selective removal of materials through wet etching for aluminum or $SF_{6}$ reactive ion etching (RIE) for niobium.

For the fabrication of the Josephson junctions, electron beam lithography was employed, utilizing a 30 kV electron beam and the Dolan technique on a Raith E-Line Plus. The chosen bottom resist was a co-polymer, specifically AR-P 617.08 (MMA), from Allresist GmbH located in Strausberg, Germany, known for its sensitivity being 3 to 4 times greater than that of PMMA resists. It was applied as a 500 nm coating by spin-coating at 4000 rpm. The top e-beam resist selected was PMMA 950 k; the optimal thickness, achieved by selecting AR-P 672.045, was a 230 nm thick layer when also spun at 4000 rpm. Each resist layer was subjected to a baking process—the first at 200 °C and the second at 180 °C, each for 10 min—to ensure the integrity of the resist structure for the Josephson junction. For room-temperature measurements, we employed a lock-In amplifier coupled with contact needle probing. Measurements in the millikelvin range were performed using a BLUEFORS, LD400 Dilution Refrigerator, Brooklyn, NY, USA.

The Josephson junctions were fabricated through a single e-beam lithography process followed by a standard Dolan-bridge double-angle evaporation of aluminum in a dedicated ultra-high-vacuum (UHV) deposition system [25]. This system, crucial for finely adjusting the tunneling barrier and preventing contamination, operates under conditions that significantly enhance the quality and reproducibility of the junctions. A detailed description of sample fabrication and techniques used is provided in the appendix. Briefly, the fabrication process begins with e-beam exposure, followed by development in MIBK:IPA 1:3 and rinsing in isopropyl alcohol (IPA) to stop the development process, then gently drying with N_2_. The samples are subsequently placed in the deposition chamber for thin film deposition and oxidation. The first step involves depositing ultrapure (99.999%) Al at a 30° angle relative to the sample’s normal (Figure 1c) using e-beam vapor deposition in a UHV environment ($10^{-9}$ Torr) to minimize impurities and achieve anisotropic deposition by positioning the sample approximately a meter away from the crucible. Following the first deposition, the sample is moved to a separate chamber and exposed to an $O_{2}$ atmosphere at 5.7 Torr to form a controlled oxide barrier (Figure 1d). Finally, the sample is returned to the main chamber for a second Al deposition at an angle of −30° (Figure 1e), completing the fabrication process.

Following the deposition process, the sample is subjected to a lift-off procedure, uncovering the structures depicted in Figure 1f. The thin oxide barrier formed during deposition exhibits a notable variation in characteristic resistance at room temperature, which can range from a few Ohms to tens of kOhms, depending on the junction’s area and size. This variation in room temperature resistance is directly proportional to the critical current value below the superconductor’s critical temperature. By optimizing this resistance, it is possible to finely adjust the performance of these devices, enhancing their functionality and efficiency in superconducting circuits.

Achieving consistent resistance measurements for multiple junctions at room temperature necessitates adherence to a meticulously optimized fabrication protocol. Despite such rigorous optimization, the inherent physical variations on the fabrication process can still lead to fluctuations in resistance measurements [26], highlighting the fragile nature of this method and underscoring the imperative need for solutions aimed at enhancing reproducibility, emphasizing the importance of both precision in the fabrication process and the pursuit of innovative strategies to ensure the reliability of Josephson junctions.

Having delineated the fabrication process, we now turn our attention to the optimization of electron beam lithography (EBL) through the simulation of electron trajectories. To elucidate the interaction between the electron beam and our sample, we employed Casino [31,32] software from Department of Electrical Engineering and Computer Engineering - University of Sherbrooke, Canada, for simulating the trajectories of electrons. Utilizing the Monte Carlo method for these simulations allows us to integrate the findings directly into our fabrication strategy. In the software, we define a bi-layer resist over a Si substrate and their respective material properties. The initial layer is composed of an MMA co-polymer, with a density of 0.80 g/cm^3^ and a thickness of 500 nm, followed by a second layer of PMMA, with a density of 1.14 g/cm^3^ and a thickness of 230 nm. The chosen substrate is silicon (Si), with a density of 2.33 g/cm^3^. Data were gathered through the simulation of 2 million electron trajectories, employing a beam radius of 10 nm and beam energy of 30 kV.

## 3. Results

**Figure 2:**
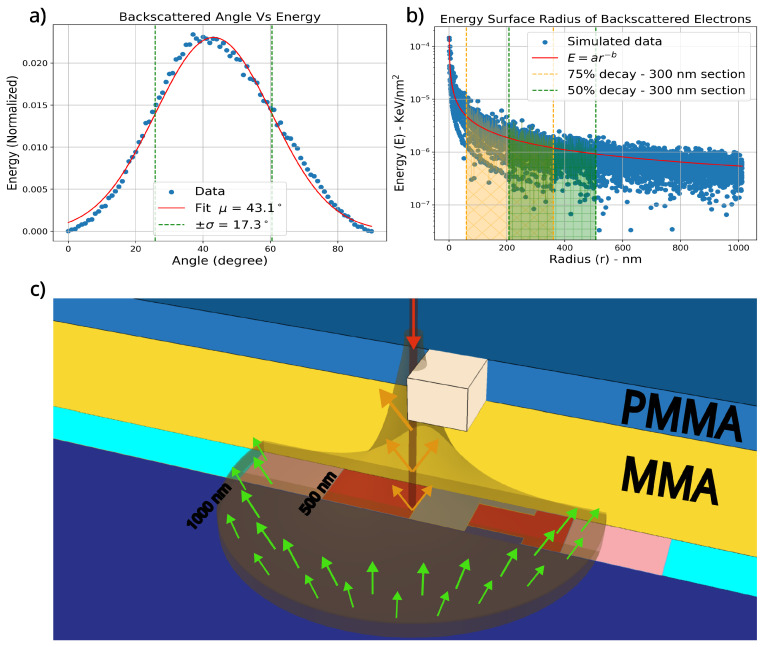
Statistical analysis of simulated backscattered electron trajectories, illustrating the correlation with angle and radius, accompanied by a schematic representation of the energy surface. (**a**) Simulated backscattered energy versus angle, with the green dashed line indicating the 2σ confidence region. (**b**) Radius of the deposited energy surface for backscattered electrons as determined by simulation. First 300 nm section to be within the resist-material-selectivity threshold is from 60 to 360 nm, where the fitted backscattered energy will decay 75% from start to end, and this region is shaded in orange. The comparative 50% decay region is shaded in green. (**c**) The energy surface of the backscattered electrons (orange shade) surrounding the incident beam (red), integrated into a cross-sectional diagram of the bridge region. The pattern areas within 500 nm of the bridge section are highlighted in red, and areas within 1000 nm are shown in light red. The red arrow depicts the beam incident direction, the orange arrow shows backscattered electrons within the resist stack, and the green arrows indicate backscattered electrons permeating from within the substrate.

To elucidate the role of backscattered electrons, the distribution of energy was analyzed with respect to both the scattering angle, as depicted in Figure 2a, and the radius of energy distribution, as shown in Figure 2b. The analysis revealed that the scattering angle’s energy deposition is best modeled by a normal distribution, with the most probable scattering angle centered around $\mu \approx 43\pm 17^{\circ }$. However, electron scattering occurs throughout the beam’s path, necessitating an examination of the cumulative effect of this scattering, represented by the radius of the backscattered energy surface. By fitting the data to a power-law decay, described by the relationship $\mathrm{Energy}=a\cdot {\mathrm{Radius}}^{-b}$, we derived the equation E(r) = $1.13\times 10^{-4}\cdot r^{-0.77}$ to characterize the energy distribution’s decay. The analysis of this distribution indicates that while incident electrons exhibit a well-defined resolution, backscattered electrons influence a broader region. This observation aligns with expectations, as the electron beam is focused down to a 10 nm radius and is subsequently dispersed in all directions upon scattering, giving origin to the backscattered electrons.

The observed decay profile stems from longer wavelengths inherent in low-energy electron beam lithography, leading to superficial scattering effects. The implications on patterning can be determined through a comparison of this decay profile with the dimensions of the features targeted for patterning. Farther from the beam, distribution becomes approximately constant. We show this effect by plotting the backscattered energy surface distribution in three dimensions as a simplified model, aligning it with the cross-section of the exposure pattern diagram. This method qualitatively displays the cumulative energy distribution’s decay along the junction area, as shown in Figure 2c.

The analysis of dose deposition by backscattered electrons provides crucial insights into the challenges associated with applying doses near the unexposed bridge region, which can lead to significant deformation due to the uneven spread of the applied dose. To mitigate the need for large doses in the vicinity of the junction area, it is essential to design geometries that strategically enhance the incidence of backscattered electrons. This approach aims to administer smaller, more uniform doses to the resist stack in the junction area from distributed regions. By comparing different geometries, we demonstrate how achieving a uniform dose distribution, or “saddle homogeneity,” is key to enhancing the robustness of the process against variations.

**Figure 3:**
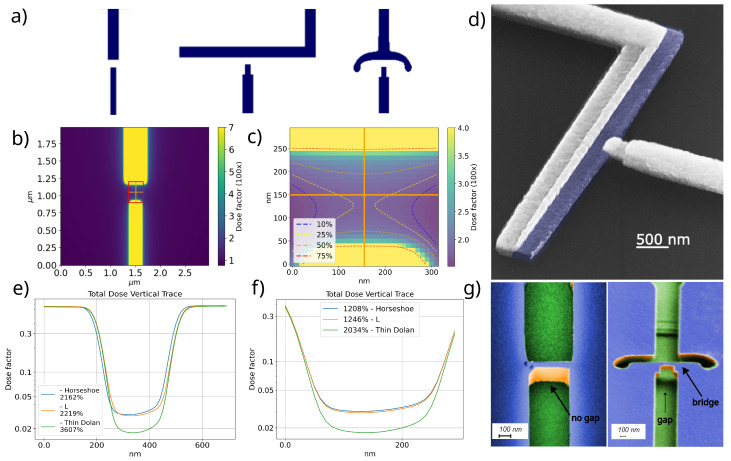
(**a**) Designs employed to investigate the effects of backscattered electrons across different geometries, identified from left to right as thin Dolan, L, and horseshoe junction designs. (**b**) Resulting dose map from the integration of the Point Spread Function (PSF) over the thin geometry, with detailed analysis presented in panels (**e**,**f**). (**c**) Detailed view of the unexposed bridge region, with percentiles marking the total deposited dose per region. (**d**) Colored scanning electron microscope (SEM) image of an L-shaped Josephson junction, where the blue region indicates the first deposited aluminum layer and the $AlO_{x}$ tunneling barrier is highlighted in the center. (**e**) Total dose distribution profiles along the vertical trace, including some of the exposed region (200 nm on each side), (**f**) only 300 nm unexposed section, for horseshoe, L, and Thin Dolan geometry—percentiles here denote the range of maximum dose variation; the percentages on the legend in (**e**,**f**) are the ratio of energy deposited in the directly exposed areas to the indirectly exposed gap. (**g**) Angled colored SEM images showcasing the resist stack; on the left, the Thin Dolan pattern is inscribed without bridge formation, whereas on the right, the horseshoe pattern is exposed, clearly displaying the bridge structure. Green indicates Si substrate, blue PMMA resist surface, and orange for resist side walls seen at an angle.

To establish a reference for tolerance to variations in the fabrication process, we selected three different geometries to evaluate the dose-dependent room-temperature resistance. The tested geometries are illustrated in Figure 3a. We fabricated multiple samples with doses varying progressively from underdoses to overdoses, adjusting the design using proximity correction software. Standard test pads were created to minimize variations in process parameters, ensuring all junctions were produced within the same chip, subject to the same conditions. We applied a varying dose from 350 to 870 μC/cm^2^ at 20 μC/cm^2^ intervals. Each geometry started with a dose low enough to reveal the exposure pattern but not high enough for the bridge structure to form, resulting in a measured resistance equivalent to an open circuit. As we proceed to measure junctions exposed to higher doses, the bridge structure begins to form, and a measurable resistance emerges. At first, resistance is very high because the bridge gap is small. This indicates the first junction measurements begin with the highest possible resistance and smallest junction area. As the dose increases, it wears down both the PMMA and MMA, increasing the dose in the unexposed areas and widening the gap, leading to a decrease in the measured resistance. Once the dose deposited in the bridge region is high enough to compromise the PMMA bridge structure, it breaks and ceases to cast a shadow, allowing the top aluminum layer to form a closed circuit, culminating in a short measurement. In this manner, we defined a dose window starting from the first junction, which resulted in a measurable resistance to the last.

To analyze the results, we present in Table 1 the dose window within which measurable resistance was obtained for each analyzed geometry. For these geometries, the dose range for which a bridge structure forms is 260 ± 10 μC/cm^2^ for the horseshoe and 160 ± 10 μC/cm^2^ for the L junction. In contrast, the thin Dolan structure exhibits stability within a narrower span of only 20 ± 10 μC/cm^2^, implying that only one dose resulted in a measurable resistance. Such a limited dose window for the thin Dolan design significantly reduces the process’s success rate. Minor changes in temperature or development time could render the samples unusable. Furthermore, we see that Horseshoe reproducibility is 96 percent while the L junction is 83 percent. Although there is a notable difference in the standard deviation, the process was repeated in different facilities with similar reproducibility. We attribute this difference to the properties of the local EBL systems.

To gain insight into the dose distribution across the junction area, we employed the Point Spread Function (PSF) [33], by using the distributions derived from Monte Carlo simulations (Casino) and applying a MATLAB 2016 open source package Urpec [34]. We then integrated PSF over the exposed geometries using a step size of 10 nm, we calculated the deposited dose within a 10 × 10 μm^2^ area; a close-up is depicted in the dose distribution plot in Figure 3b. A detailed examination of the unexposed bridge region is showcased in Figure 3c, where orange lines represent vertical and horizontal traces without exposure. Further analysis of these traces enabled us to characterize the distribution of the total applied dose across various geometries and conditions. The noted asymmetry in the saddle-shaped energy distribution can be ascribed to one side of the junction being wider, facilitating a seamless contact between the upper and the oxidized (purple) lower layers of the Josephson junction, as demonstrated in the SEM angled image of the L-type junction (Figure 3d), where the upper layer is observed making a clean and smooth contact with the lower layer.

To understand why the L and horseshoe are reproducible while the thin Dolan design is not, we begin by examining the ratio of energy deposited in the directly exposed areas to the indirectly exposed gap, shown in Figure 3e. From this graph, we can see that the exposed regions are subject to the same dose; however, there is a sharp decrease in total deposited dose from 0.6 to less than 10% of this value for all geometries. A more detailed examination of the unexposed region, shown in Figure 3f, indicates the total dose within 300 nm of the bridge section changes approximately 12 times for the most effective geometries, the horseshoe, and the L, and 20 times for the thin Dolan, which exhibits a lower success rate. Assuming the same dose is necessary for gap formation, the thin Dolan structure necessitates a higher geometry dose factor to modify the solubility of the MMA layer, leading to pattern formation before the bridge structure, which is noticeable in the SEM image in Figure 3g (left). This implies that a higher dose will also be deposited on the top PMMA layer, which is undesirable, as it weakens the bridge structure, causing some to break while others become narrower, thus reducing the reproducibility of Josephson junctions. To mitigate this issue, we investigate geometry-dependent designs to explore how the backscattered electron distribution selectively modifies the solubility of the MMA resist.

**Figure 4:**
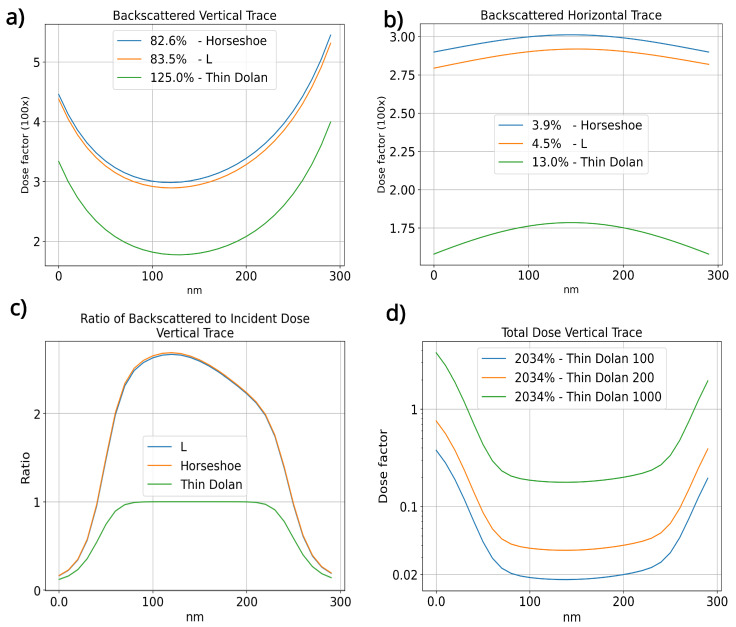
Analysis of dose factors and the ratio of the backscattered to total incident dose for various geometries investigated in this study. (**a**) Distribution of the backscattered electrons along a vertical trace. (**b**) Distribution along a horizontal trace. (**c**) Ratio of backscattered to incident dose over the vertical trace. (**d**) Dose variation observed in a thin Dolan geometry.

A closer look at the disposition of only the backscattered electrons for the three geometries (Figure 4a,b), shows that the horse and L geometries have a more homogeneous backscattered electron distribution than the thin Dolan geometry. Furthermore, it can be noted that the horse and L junction have over 50% more dosage deposited over the bridge area, showing that a smaller dose near the junction area is needed to form the bridge structure, preserving the PMMA structure for the reasons stated previously. This can be seen in Figure 3g (right), where a patterned resist for the horseshoe junction has a clear gap formed.

To further understand the impact of the incident electrons, which may undergo forward scattering and drift from their point of incidence, we integrated the Point Spread Function (PSF), excluding the backscattered terms to analyze the distribution of incident energy, and calculated the ratio of deposited dose between incident and backscattered electrons, denoted as $E_{b}/E_{i}$. Figure 4c illustrates this ratio along the vertical orange line traversing the bridge area, as shown in Figure 3c. As shown by the behavior of $E_{b}/E_{i}$ in the bridge region for the three geometries observed, the contribution of backscattered electrons is at least twice as much as the dose deposited by incident electrons alone in the central part of the bridge region for the horseshoe and L-shaped designs. Meanwhile, for the thin Dolan design, this ratio in the vicinity of the bridge’s central part exhibits a comparable effect.

Figure 4d displays the total dose deposited by the incident beam in the bridge region for different base doses within the thin Dolan geometry. An increase in the base dose outside the bridge region elevates the total deposited dose within the bridge area as anticipated, but does not alter the dose ratio between the regions outside the bridge and its central area. Consequently, a higher base dose intensifies the dose deposited throughout the entire resist stack, diminishing the bridge’s stability.

## 4. Discussion

**Figure 5:**
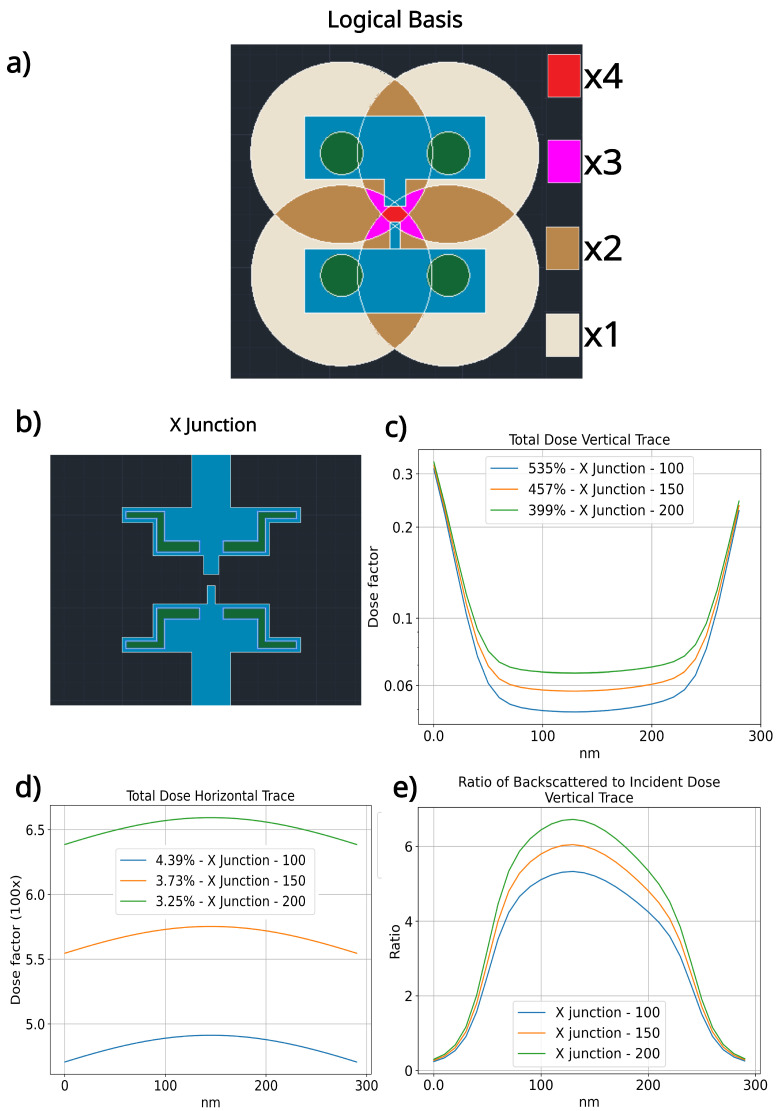
Analysis of dose factors and the ratio of the backscattered to the total incident dose for the proposed geometry designed to utilize backscattered electrons for the undercut definition. (**a**) The logical basis to create a new geometry, specifically conceived to tailor the distribution of backscattered electrons, thereby minimizing variance across the junction area. The blue region is intended to receive the minimal necessary dose to develop the top resist layer, with backscattered electrons being generated within the green circle region by a higher dose factor. The larger circles represent a simplified model for the overlap of the backscattered regions, assuming point sources, with different colors indicating the degree of overlap. (**b**) X junction ggeometry designed with features to retain geometric resolution while achieving the (4:1) ratio for the (**c**) total deposited dose over the vertical trace. (**d**) Horizontal profile of the total deposited dose. (**e**) Ratio of backscattered to incident dose for the X junction.

The simulations indicate that an optimized geometry should have enough exposed area within a 4 μm radius of the junction area to eliminate the need for increased dose around the region of the bridge, which can cause the PMMA bridge structure to deteriorate. As a means to elucidate our findings pragmatically, we propose an innovative e-beam lithography technique in two steps. We suggest the geometry should be optimized in resolution first rather than undercut. Once a a base dose for the geometry is found, strategic places are used to engineer the correct backscattered electron dose. A schematic of such a configuration is depicted in Figure 5a. The blue region receives the constant base dose, while the four green zones receive higher doses to create backscattered electrons. If sources were point-based and equidistant from the junction area, they would overlap with maximum interference over the unexposed junction region. Based on this logic, we created four regions; however, to avoid extreme doses, we increased the area (Figure 5b). Simulations showed that by raising the dose on the backscattered electron regions, the bottom of the vertical trace is modulated while creating negligible effects on the border, making the whole length of the bridge region maintain a 400% dose variation or less (Figure 5c). It should be noted that this distribution will remain constant for this geometry as long as the dose on the backscattered electron region is twice the base dose. The horizontal trace of the bridge area is plotted (Figure 5d,e). The ratio of the backscattered to incident dose in Figure 5e is significantly higher compared to previous junctions, and this geometry allows control over this ratio, providing an efficient means to precisely control the undercut to increase overall Josephson junction reproducibility. Although it has not been tested, we believe designating zones to produce backscattered electrons is a promising means of increasing the success rate in junction fabrication. Furthermore a complete description of simulated dose distributions for the unexposed region of the junction are displayed on Table 2, where we show the total and isolated backscattered dose variation along the vertical and horizontal traces for all simulated results. Information on the contribution of sideways scattered incident electrons can be inferred from the ratios along the vertical traces. The incident dose is the main source of the dose at the very edge of the unexposed regions; however, it diminishes greatly within the first tens of nanometers, showing that for any geometry, the backscattered electrons are the main source of dose deposition at approximately the center of unexposed regions, from where the max ratio value is derived.

**Figure 6:**
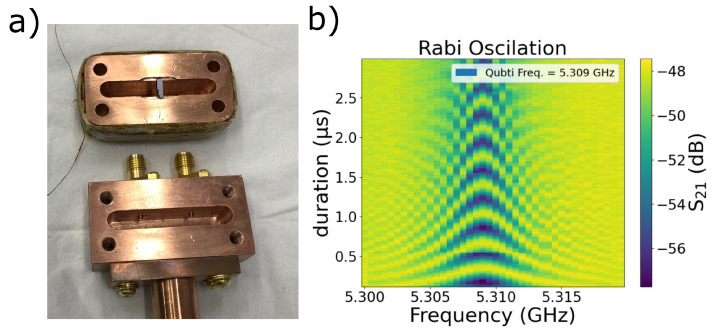
(**a**) Three-dimensional copper cavity. (**b**) Quantum Rabi map that provides a profile of the dynamic evolution of qubit states in a time-dependent landscape.

As a proof of concept, we successfully fabricated and analyzed a qubit within a (3D) cavity displayed in Figure 6a. The qubit construction involved L-shaped junctions, similar to the ones shown in the scanning electron microscope (SEM) image in Figure 3d. They were integrated into a SQUID with the rectangular en-looped area of (23 × 90) μm^2^ enclosed by two capacitive plates (300 × 800) μm^2^. This device was placed in a 3D cavity and mounted onto the cold plate of a Dilution refrigerator. The cavity and qubit frequency were measured at 7.389 GHz and 5.309 GHz, respectively. In the course of our characterization, we observed Rabi Oscillations displayed in Figure 6b and key performance metrics for the qubit. The relaxation time ($T_{1}$) was measured at 14.3 ± 0.4 μs, and the coherence time ($T_{2^{*}}$) was measured using the Ramsey protocol at 1.0 ± 0.1 μs. These characterizations provide evidence of the functionality and quality of the created Josephson junctions composing the qubit within the 3D cavity.

## 5. Conclusions

This study provides a comprehensive investigation into the fabrication of Josephson junctions using 30 kV e-beam lithography. We address the critical role of backscattered electrons in the fabrication process, emphasizing the importance of controlling their distribution. Our analysis establishes a parameter to define dose stability, which agrees with the experimentally obtained reproducibility standards of three different lithography patterns. Through simulations, we demonstrated that for conventional one-step 30 kV EBL for the Dolan technique, some geometries achieve more uniform dose deposition than others, affecting junction stability and performance. We developed a systematic means of analyzing simulation results, which allowed us to separate incident from backscattered contribution, which underscored the geometric effect on dose distribution.

As our simulations revealed, the strategic control of backscattered electrons can indeed lead to a more uniform distribution of dose deposition, distinctively showing a possible reason for some geometries to yield higher than others. In Table 2, geometries are ordered from least to most homogeneous, establishing a pattern to be followed and enabling us to propose a new geometry, with the intent of mitigating the issues raised in the article.

The results made it evident that low-energy EBL needs to especially consider the distribution of backscattered electrons. Simulation techniques, such as those detailed in this study, are instrumental in determining if a given exposure pattern incorporates sufficient backscattered contributions to create controlled undercuts. This technique is a notable contribution to not only the fabrication of Josephson junctions, but also to many other lithography processes, which require undercut engineering and control. To highlight our conclusions, we provided a geometric example that has built-in regions used strategically for backscattered electron generation. These zones are used to apply a controlled homogeneous dose over the junction area, mitigating the challenges associated with the fabrication of bridge-like structures. While the effectiveness of this geometry has not been tested, it serves as a possible direction for continued research. Josephson junctions are devices with great innovative potential, and our research democratizes their production, making it more accessible to fabricate Josephson junctions reliably using 30 kV EBL systems.

## Figures and Tables

**Table 1:** Experimentally defined reference for process tolerance per geometry. Reproducibility standards for geometries that have been thoroughly tested. The standard deviation for room temperature resistance.

Junction Type	Supported Dose Variation (μC/cm^2^)	Reproducibility	Std Deviation
Thin Dolan	20 ± 10	Not reproducible	-
L shape	160 ± 10	83% (20/24)	3.2%
horseshoe	260 ± 10	96.3% (26/27)	31.7%

**Table 2:** Table displaying simulated dose variation in the unexposed horizontal and vertical traces of the junction area. Variations along traces are calculated by dividing the maximum by the minimum dose value. The max ratio is defined by comparing the backscattered to incident dose along the vertical trace.

	Total Vertical	Total Horizontal	Backscattered Vertical	Backscattered Horizontal	Max Ratio
Thin Dolan	2034%	13.0%	125.0%	13.0%	1
L	1246%	11.1%	83.5%	4.5%	2.7
Horseshoe	1208%	3.9%	82.6%	3.9%	2.7
X Junction (100)	535%	4.4%	37.3%	4.7%	5.3
X Junction (150)	457%	3.7%	32.0%	3.7%	6.1
X Junction (200)	399%	3.3%	28.1%	3.3%	6.7

## Data Availability

Data available within the paper or via reasonable request.

## Abbreviations

The following abbreviations are used in this manuscript:

PEC	Proximity Effect Correction
UHV	Ultra-High Vacuum
EBL	Electrom Beam Lithography
PMMA	Poly(methyl methacrylate)
MMA	Methyl Methacrylate
IPA	Isopropyl Alcohol
